# Coplanar-gate ZnO nanowire field emitter arrays with enhanced gate-control performance using a ring-shaped cathode

**DOI:** 10.1038/s41598-018-30279-y

**Published:** 2018-08-16

**Authors:** Long Zhao, Yicong Chen, Zhipeng Zhang, Xiuqing Cao, Guofu Zhang, Juncong She, Shaozhi Deng, Ningsheng Xu, Jun Chen

**Affiliations:** State Key Laboratory of Optoelectronic Materials and Technologies, Provincial Key Laboratory of Display Material and Technology, School of Electronics and Information Technology, Sun Yat-sen University, Guangzhou, 510275 People’s Republic of China

## Abstract

Nanowire field emitters have great potential for use as large-area gated field emitter arrays (FEAs). However, the micrometer-scale cathode patterns in gated FEA devices will reduce regulation of the gate voltage and limit the field emission currents of these devices as a result of field-screening effect among the neighboring nanowires. In this article, a ring-shaped ZnO nanowire pad is proposed to overcome this problem. Diode measurements show that the prepared ring-shaped ZnO nanowire pad arrays shows uniform emission with a turn-on field of 5.9 V/µm and a field emission current density of 4.6 mA/cm^2^ under an applied field of 9 V/µm. The ZnO nanowire pad arrays were integrated into coplanar-gate FEAs and enhanced gate-controlled device characteristics were obtained. The gate-controlled capability was studied via microscopic *in-situ* measurements of the field emission from the ZnO nanowires in the coplanar-gate FEAs. Based on the results of both simulations and experiments, we attributed the enhanced gate-controlled device capabilities to more efficient emission of electrons from the ZnO nanowires as a result of the increase edge area by designing ring-shaped ZnO nanowire pad. The results are important to the realization of large-area gate-controlled FEAs based on nanowire emitters for use in vacuum electronic devices.

## Introduction

Large-area gated field emitter arrays (FEAs) are important for applications in vacuum electronic devices such as flat panel X-ray sources, imaging detectors, field emission displays (FEDs) and light sources^1–6^. For example, because each of the pixels of a flat panel X-ray source can be controlled independently, the emission area of the flat-panel X-ray source can be selectable, and thus only the area of interest is irradiated. A flat-panel X-ray source based on an addressable pixel array of cold cathodes could therefore reduce the dose required for X-ray inspection dramatically^7,8^. The development of large-area addressable FEAs is thus of major significance for the realization of such applications.

Because of their high aspect ratios, one-dimensional nanostructures have been explored for FEA applications^9^. Among the materials studied to date, ZnO nanowire emitters show promising application as field emitters due to their excellent field emission performances^10,11^. ZnO nanowires can be synthesized by various methods, including thermal evaporation/condensation^12^, chemical vapor deposition^13^, hydrothermal solution^14^, and thermal oxidation^15^, which indicate the feasibility of integration of these nanowires in device structures^16,17^. In particular, ZnO nanowires can be synthesized from a zinc thin film under mild temperature via a simple thermal oxidation method. The thermal oxidation process is compatible with glass substrate and is scalable to large area, which have great application potential in large area gated FEAs.

When compared with Spindt-type molybdenum FEAs or silicon FEAs, FEAs based on ZnO nanowire pad emitters have advantages that include a simple preparation process, and uniform large-area production capability. However, conventional photolithography methods can only be used to produce micrometer-scale pads; while the nanowires at the edge of such a pad will be regulated strongly by the gate, the remaining nanowires on the pad are only weakly controlled because of the field-screening effect that occurs among the neighboring nanowires^18,19^, and this can reduce regulation of the gate and limit the field emission current of the nanowire pad. Therefore, enhancement of the regulation of the gate voltage to improve the field emission current would be one way to improve the performance of the resulting gated nanowire FEAs.

In this work, a ring-shaped ZnO nanowire pad structure is proposed with the intention of providing enhanced gate voltage regulation. We first study the field emission characteristics of the proposed ZnO nanowire pad arrays using a diode structure. We then investigate the emission properties of the coplanar-gate ZnO nanowire FEAs when used in a FED. Finally, we investigate the origins of the efficient field emission from the proposed ring-shaped ZnO nanowire pads through simulations. Additionally, microscopic *in-situ* field emission measurements of the ZnO nanowires in various regions of the ring-shaped ZnO pad in the coplanar-gate FEA under various gate voltages were conducted in an ultrahigh vacuum system and the results verified our proposed structure.

## Methods

### Design of a gated structure and shape of the ZnO nanowire pad

**Figure 1:**
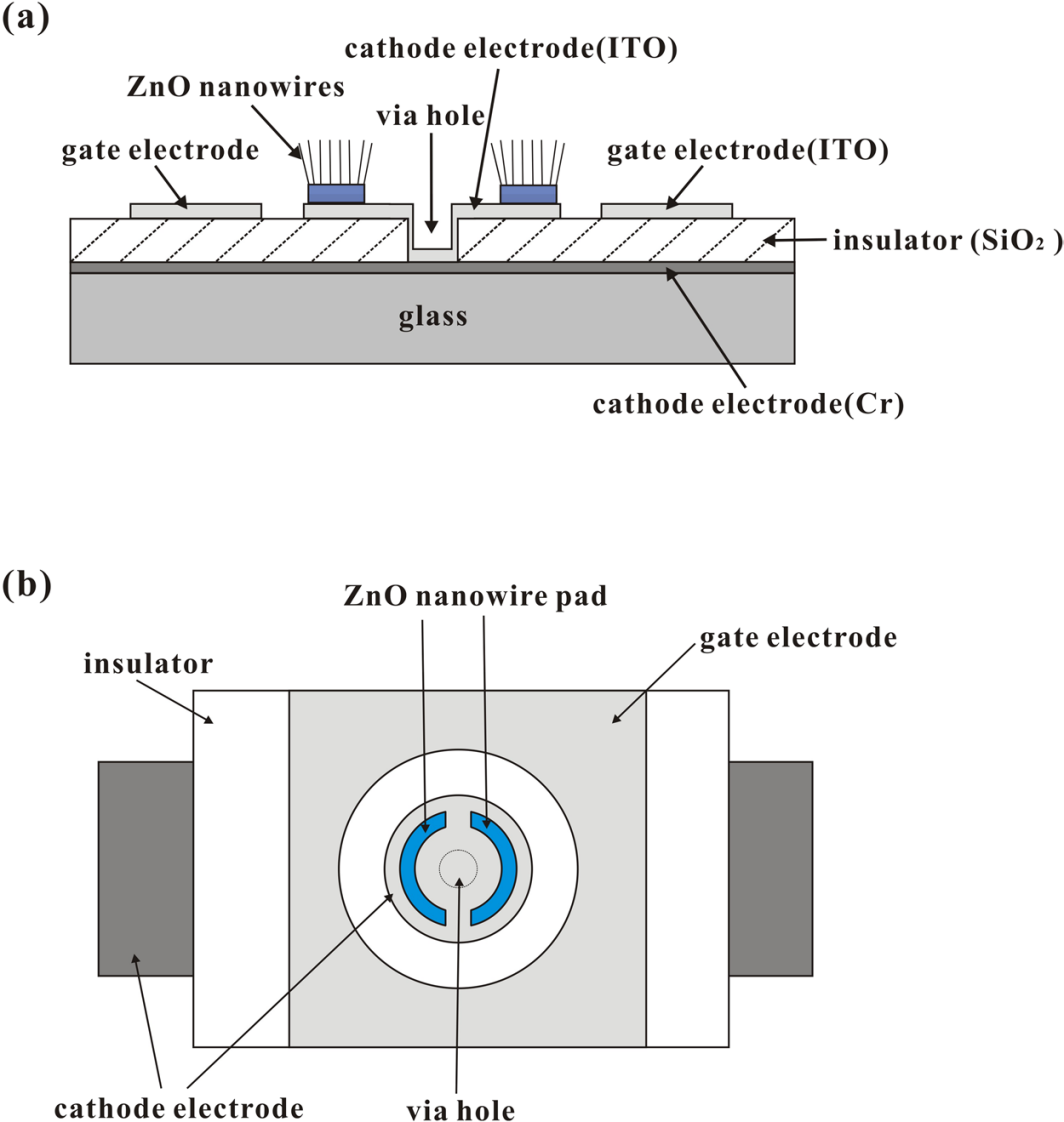
Schematic diagrams of the coplanar-gate ZnO nanowire FEA structure: (**a**) cross-sectional view; (**b**) top view.

We designed an applicable gated structure to incorporate the proposed ZnO nanowire field emitters. A schematic of the coplanar-gate ZnO nanowire FEA structure is shown in Fig. 1. Figure 1(a) shows a cross-sectional schematic diagram of the structure, in which the gate electrodes are located around the cathode electrodes and the electrodes are separated by an insulating layer. The coplanar gate is located close to the ZnO nanowire pads aiming to reduce the gate driving voltage. Figure 1(b) shows that a ZnO nanowire pad shape that consists of two narrow half-rings is used. This design is intended to increase edge area of the ZnO nanowire pad and suppress the diode effect in the gated structure (see Supplementary Fig. S1). The Zn films were patterned using standard photolithography methods and lift-off processes. The shape with two half-rings rather than a full ring was adopted for ease of fabrication and helps to avoid Zn film peel-off during the lift-off process. The symmetrical structures of our designed ZnO nanowire pad and gate electrode also ensure uniform electric field distributions around the ZnO nanowire pad and produce uniform emission sites.

### Preparation of the ZnO nanowire arrays and field emission characterization

The ZnO nanowire arrays were prepared by thermally oxidizing Zn film patterns. First, 100 mm × 90 mm soda-lime glass substrate was cleaned ultrasonically in sequence in acetone, ethanol, and deionized water for 30 min in each liquid. Subsequently, 300-nm-thick indium tin oxide (ITO) thin film was deposited by magnetron sputtering on the soda-lime glass substrate to act as the cathode electrode. Second, after the series of ultrasonic cleaning procedures described above, 1.8-µm-thick photoresist layer was spin coated on the substrate and it was then exposed to ultraviolet light in a mask aligner machine. After development, the patterned photoresist layer was obtained. Third, the substrate was bombarded *in situ* for 30 min by Ar ions that were generated using a Huffman ion source in a vacuum chamber. The pressure during the ion bombardment was approximately 5 × 10^−2^ Pa. During the bombardment process, the Ar gas flow rate was 6 sccm, the anode voltage was 215 V, and the ion beam current was 50 mA. Subsequently, 1.7-µm-thick zinc thin film was deposited by electron beam deposition and patterned using a lift-off process. Finally, the sample was placed into a horizontal quartz tube furnace in air, and the temperature was raised from room temperature to 470 °C and maintained for 3 h. When the furnace was cooled to room temperature, the ZnO nanowire arrays were prepared successfully. There are 80 × 80 ZnO nanowire pads in the ZnO nanowire arrays, where each pad consists of two 8-µm-wide half-rings. The distance between each pair of pads is 450 μm.

The top-view and cross-sectional morphologies of the prepared nanowires were then characterized using a scanning electron microscope (SEM; SUPRA-60). Photoluminescence (PL) measurement was performed by Raman spectroscopy (Renishaw Invia Reflex) using laser excitation at 325 nm. The field-emission characteristics of the ZnO nanowire arrays that were grown on the glass substrate was then measured in a vacuum chamber using a diode set-up. An ITO glass surface coated with a green phosphor was used as the anode plate, and the cathode and anode plates were kept apart using 0.5-mm-thick spacers. The voltage applied between the two plates was varied and the field emission current was recorded automatically using a computer that was connected to the current meter. The vacuum chamber pressure was maintained at approximately 3 × 10^−5^ Pa for all measurements.

### Fabrication and characterization of the gated ZnO nanowire FEAs

The gated ZnO nanowire FEA fabrication process is described as follows. First, a 120-nm-thick chromium thin film was deposited on the glass substrate by magnetron sputtering and patterned as bottom cathode electrodes via a lift-off process. Second, a 1.5-μm-thick silicon oxide film was deposited by plasma enhanced chemical vapor deposition (PECVD) to act as an insulator layer. This insulating layer was then etched by a reactive ion etching (RIE) process to produce via holes. Third, the gate electrodes and top cathode electrodes were formed together by a single mask process from a sputtered 300-nm-thick ITO thin film. The top cathode electrodes were connected to the bottom cathode electrodes through the via holes. Finally, a 1700-nm-thick zinc thin film was deposited and ZnO nanowires were grown via a thermal oxidation process. In this study, the ZnO nanowire FEA with an area of 36 mm × 36 mm was fabricated on a glass plate with an area of 100 mm × 90 mm. There are 80 × 80 ZnO nanowire pads in these FEAs, where each pad consists of two 8-µm-wide half-rings with a diameter of 60 μm on the cathode electrode. Each pixel is 450 μm × 450 μm in size. The distance between the cathode electrode and the planar gate is 10 μm.

The top-view and cross-sectional morphologies of the prepared coplanar-gate ZnO nanowire FEAs were characterized using the SUPRA-60 SEM. The field emission measurements of the ZnO nanowires in the different ZnO pad regions in the gated FEAs were performed under various gate voltages in a Nanoprobe system (Omicron Nanoscience) at a base pressure of 10^−10^ mbar. A tungsten tip that serves as the anode was placed on top of the ZnO nanowires in various regions of the ZnO pad and two Keithley 2450 power supplies were used to supply the anode voltage and the gate voltage. A Keithley 2657 current-voltage analyzer was used to measure the field emission characteristics and a Keithley 2450 power supply was used to control the gate bias (*V*_g_) of the field emission display device.

## Results

### Morphologies of the ZnO nanowire pads and photoluminescence characterization

**Figure 2:**
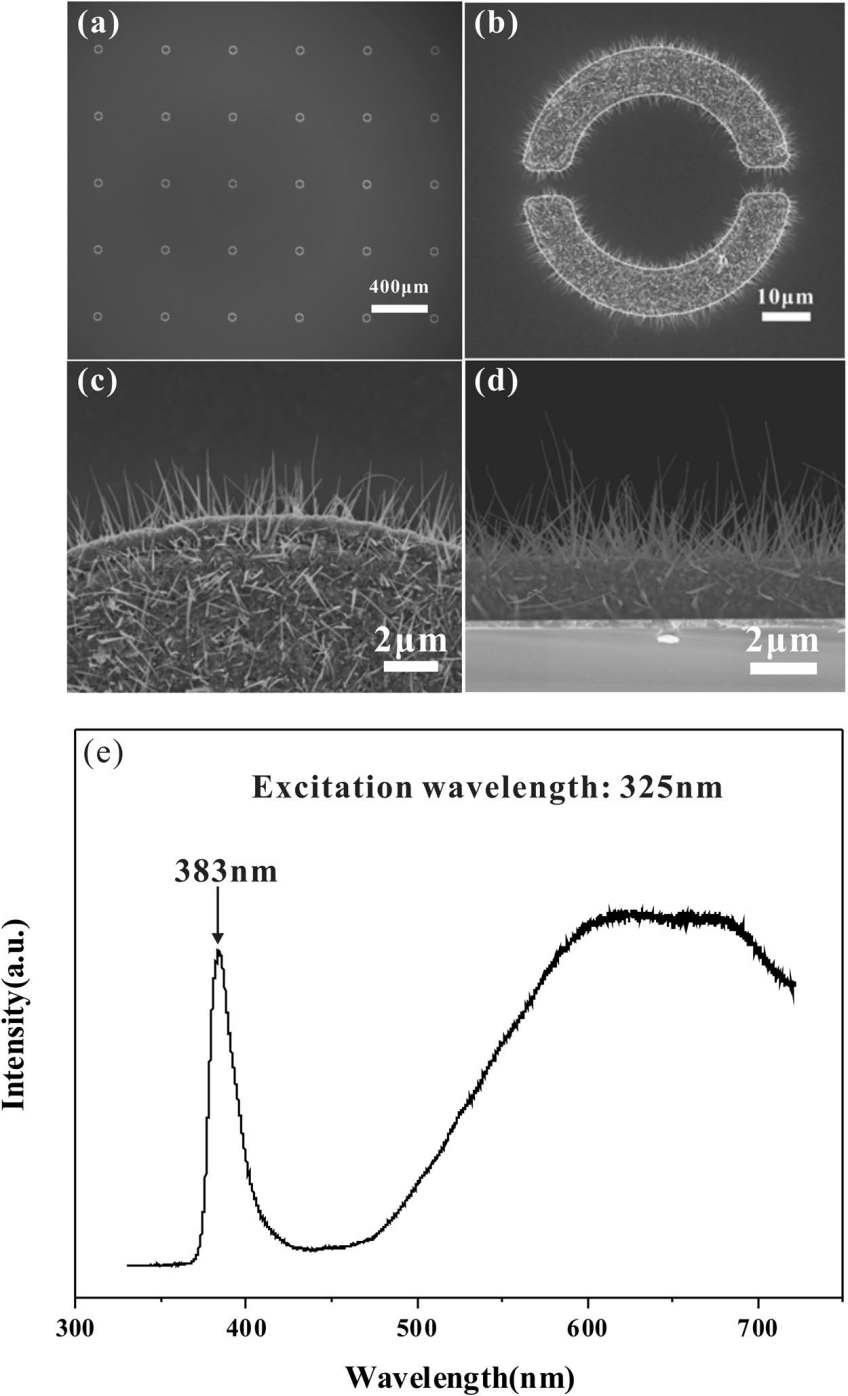
(**a**) Top-view SEM images of the ZnO nanowire pads in the array; (**b**) morphology of ZnO nanowires grown from a single Zn thin film pad; (**c**) amplified SEM image of the local region of a half-ring structure in a single ZnO film pad; (**d**) cross-sectional SEM image of the ZnO nanowire pad; (**e**) room-temperature PL spectrum of the ZnO nanowire pad in the array.

Figure 2 shows top view and cross-sectional view SEM images of the ZnO nanowires in the array and the photoluminescence (PL) spectrum of the ZnO nanowire pad in the array. Figure 2(a) shows a top-view SEM image of ZnO nanowire pads in the array where each of the ZnO nanowire pads is apparently uniformly distributed in the array. Figure 2(b) shows the morphologies of ZnO nanowires that were grown from a single zinc thin film pad. We see that dense ZnO nanowires grow on the surface and on the edges of the ZnO film. Figure 2(c) shows an amplified SEM image of a local region of a half-ring in a single ZnO film pad. Most of the ZnO nanowires clearly form nanosheets. A single ZnO nanowire has a sharp tip and a comparatively wide base. These ZnO nanowires are grown randomly on the rough ZnO film. The density of the ZnO nanowires is estimated to be 2.3 × 10^8^ cm^−2^. Figure 2(d) shows a cross-sectional SEM image of ZnO nanowire pad, it indicates that the ZnO nanowires are grown vertically and uniformly on the ZnO film. The average ZnO nanowire height is in the 3–4 μm range. The grown ZnO nanowires all have tip diameters of ∼20 nm.

The PL spectrum of the ZnO nanowire pad in the array was measured at room temperature, with result as shown in Fig. 2(e). The measurement information mostly comes from the ZnO layer, while little comes from the ZnO nanowires themselves because the influence of these ZnO nanowires is limited to the nanoscale. Two emission peaks located in the ultraviolet (UV) and visible ranges are observed in the PL spectrum and correspond to near-band-edge emissions and deep level (DL) emissions, respectively. It has previously been reported that the UV emission band arises from near band-edge transitions of ZnO with its wide band gaps^20,21^ and the broad emission band observed in the visible region is the result of structural defects (such as singly ionized oxygen vacancies)^22,23^. Figure 2(e) shows that the strength of the DL emission peak in the PL spectrum is stronger than the UV emission peak, which indicates that larger numbers of oxygen vacancies or other structural defects are generated within the ZnO layer. A higher concentration of oxygen vacancies is likely to create many electron carriers and the conductivity of the ZnO pad will thus be enhanced^24,25^.

### Field-emission characteristics of the ZnO nanowire arrays

**Figure 3:**
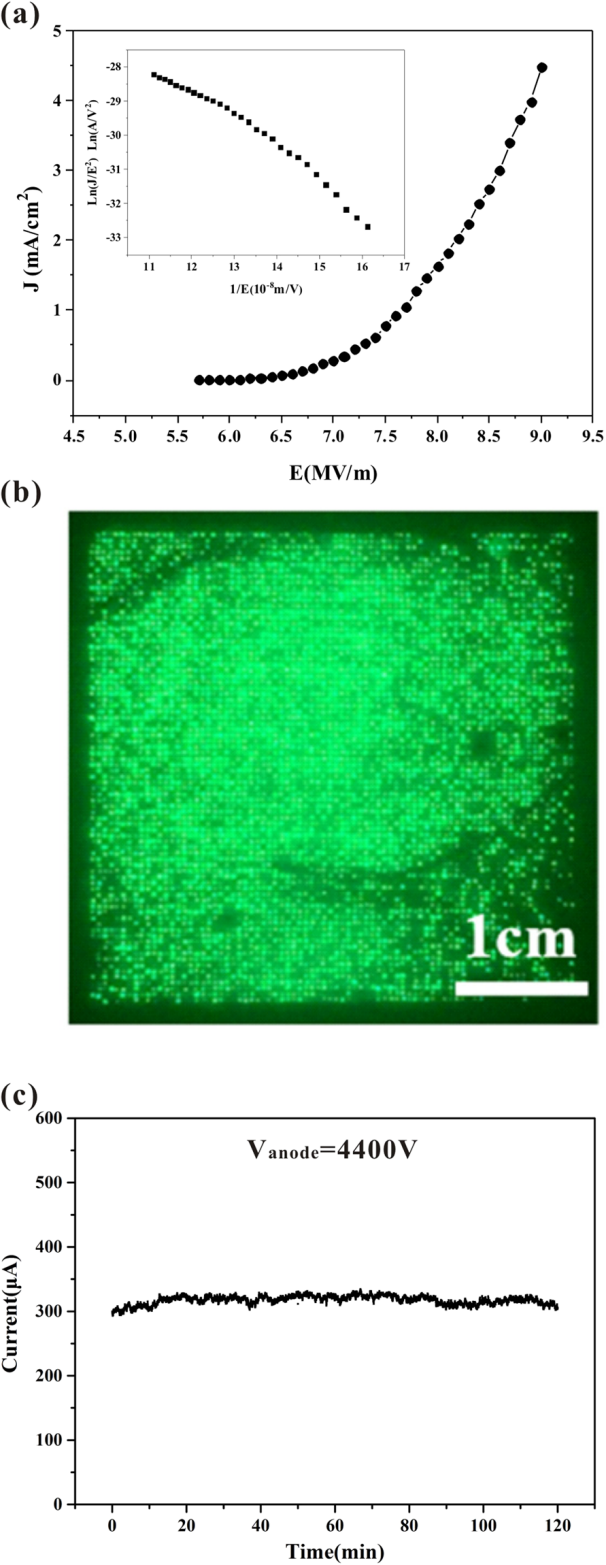
Field-emission characteristics of the ZnO nanowire arrays: (**a**) Field emission *J*-*E* curve, where the corresponding F-N plot is shown in the inset; (**b**) the field emission image; (**c**) the field emission current versus time (I-t) curve.

The field-emission characteristics of the ZnO nanowires that were prepared by the thermal oxidation method was measured. Figure 3(a) shows the field emission current density versus electric field (*J*-*E*) curve and the corresponding Fowler-Nordheim (F-N) plot. From the *J*-*E* curve, we found that the turn-on field (which is defined as the electric field at an emission current density of 10 μA/cm^2^) for the ZnO nanowire array is 5.9 MV/m. From the F-N plot shown in the inset of Fig. 3(a) exhibits a straight line, indicating that the field emission follows the classical electron tunneling mechanism. Though more complicated emission mechanism may exist for the semiconducting ZnO nanowire, the classical F-N equation can still be used for analysis of the field emission data. We therefore use the simplified F-N formulation to estimate the relative field enhancement factor. The F-N equation can be given as^26^.

1 $$J=A\frac{{\beta }^{2}{E}^{2}}{\Phi }\exp (-B\frac{{\Phi }^{3/2}}{\beta E})$$

where *J* is the emission current density (A/m^2^), *E* is the electric field (V/m), *Φ* is the work function of the emitter, and *A* and *B* are the F-N constants with values of 1.54 × 10^−6^ and 6.53 × 10^9^, respectively. The work function of ZnO is assumed to be 5.28 eV^27^. Therefore, the field enhancement factor (*β*) can be calculated based on the slope of the ln(*J*/*E*^2^) versus 1/*E* plot according to the F-N plot. The calculated *β* value of the ZnO nanowire array is 800. However, we noted that the F-N plot deviates from this linear trend when the electric field is high. We think that the semiconducting ZnO nanowire could act as a ballast resistance; this ballast resistance will then limit the field emission current when the electric field is high^1,28^.

Additionally, from the field emission (FE) characteristics measurements, the field emission image of the ZnO nanowire arrays recorded under an anode voltage of 4.5 kV is shown in Fig. 3(b). There are 80 × 80 pixels in the display region. The field emission image has a scale of 2 in (diagonal length) and an area of 3.6 cm × 3.6 cm. The observed uniform field emission image of the ZnO nanowire array was attributed to the nanowires having uniform geometries and the ballast effect of the ZnO nanowires. Figure 3(c) shows the field emission current versus time (I-t) curve of the ZnO nanowire arrays under a constant applied voltage for 2 h. The field-emission current fluctuation Δ of the ZnO nanowire arrays was calculated using Δ = (I_max_ − I_min_)/2I_ave_, where I_max_ is the maximum current, I_min_ is the minimum current and I_ave_ is the average current. The calculated current fluctuation is ~5.7%.

In summary, for the ring-shaped ZnO nanowire pad arrays that were prepared by thermal oxidation, field emission occurred at a turn-on field of 5.9 MV/m, and a FE current density of 4.6 mA/cm^2^ was produced under an applied field of 9 V/µm. A uniform emission image was also obtained.

### Application to large-area gated FEAs

**Figure 4:**
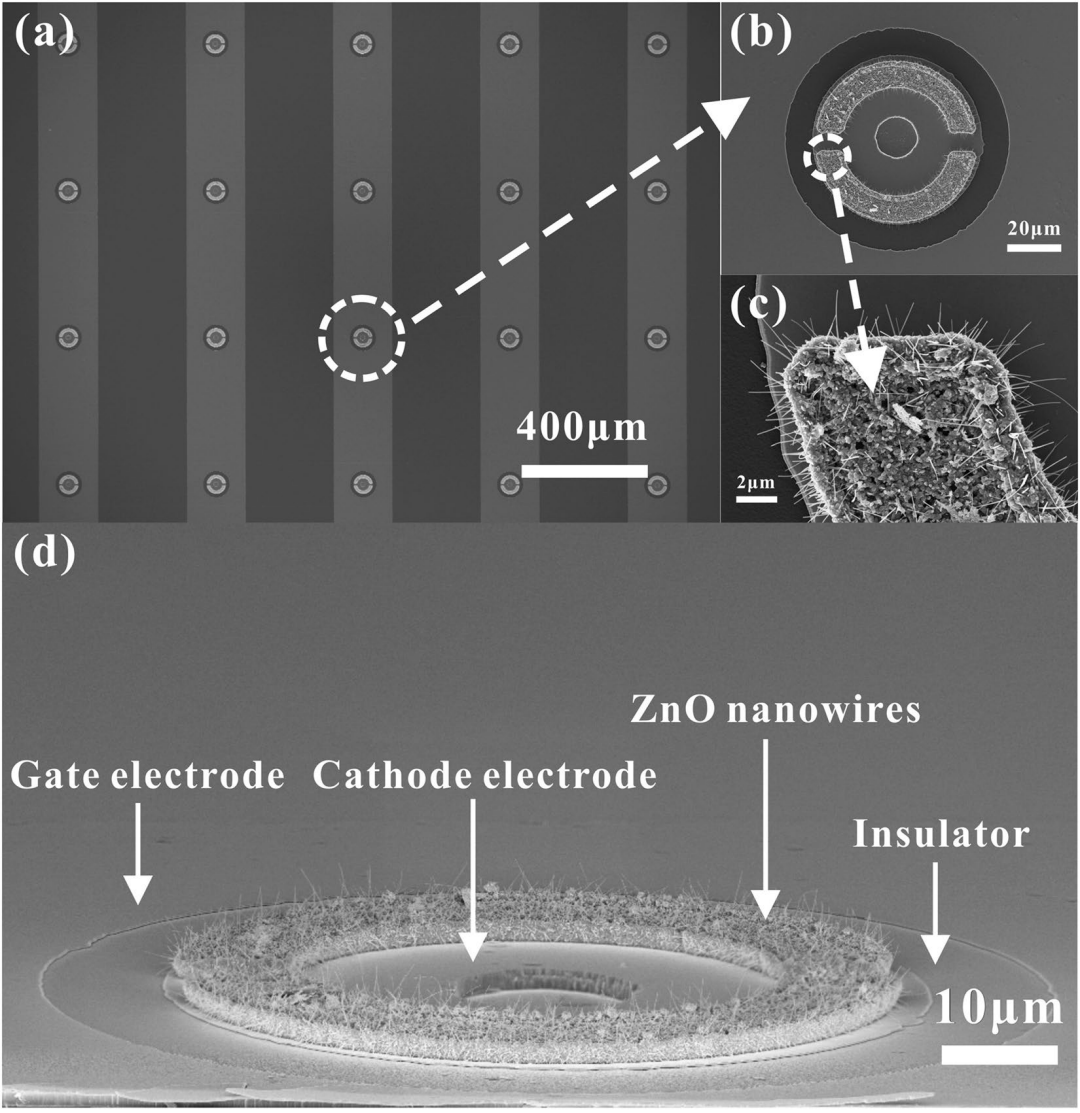
Top view and cross-sectional SEM images of the fabricated coplanar-gate nanowire FEAs. (**a**) Top view SEM image of the of gated FEA structures; (**b**) individually addressable FEA; (**c**) ZnO nanowire morphologies in the ZnO nanowire pad; (**d**) cross-sectional SEM image of the ZnO nanowire pad in an individually addressable FEA.

ZnO nanowire pads prepared by the thermal oxidation method were then integrated into gated FEAs. The fabricated coplanar-gate ZnO nanowire FEAs were observed using a SEM, and the resulting images are showed in Fig. 4. Figure 4(a) shows the top view of the fabricated coplanar-gate nanowire FEAs. The ZnO nanowire pads are distributed uniformly within the gated FEAs. Figure 4(b) shows the top view of an individually addressable FEA. The ZnO nanowire pad is centered on the cathode electrode in this image. Figure 4(c) shows the morphology of the ZnO nanowires in the ZnO nanowire pad. The ZnO nanowires in the individually addressable FEA are shown in detail in the cross-sectional SEM image in Fig. 4(d). Good compatibility was observed for the ZnO nanowires that were grown in the gated FEAs.

To enable study of the field emission characteristics of these gated FEAs, the coplanar-gate ZnO nanowire FEAs acted as cathode plate was assembled with an ITO glass substrate coated with green phosphor which acted as anode plate to form a FED. The cathode and anode plates were kept apart using 500-μm-thick spacers. The two separated plates were sealed using glass frit and the FED device’s pressure was maintained at 3 × 10^−5^ Pa using a dynamic vacuum system.

**Figure 5:**
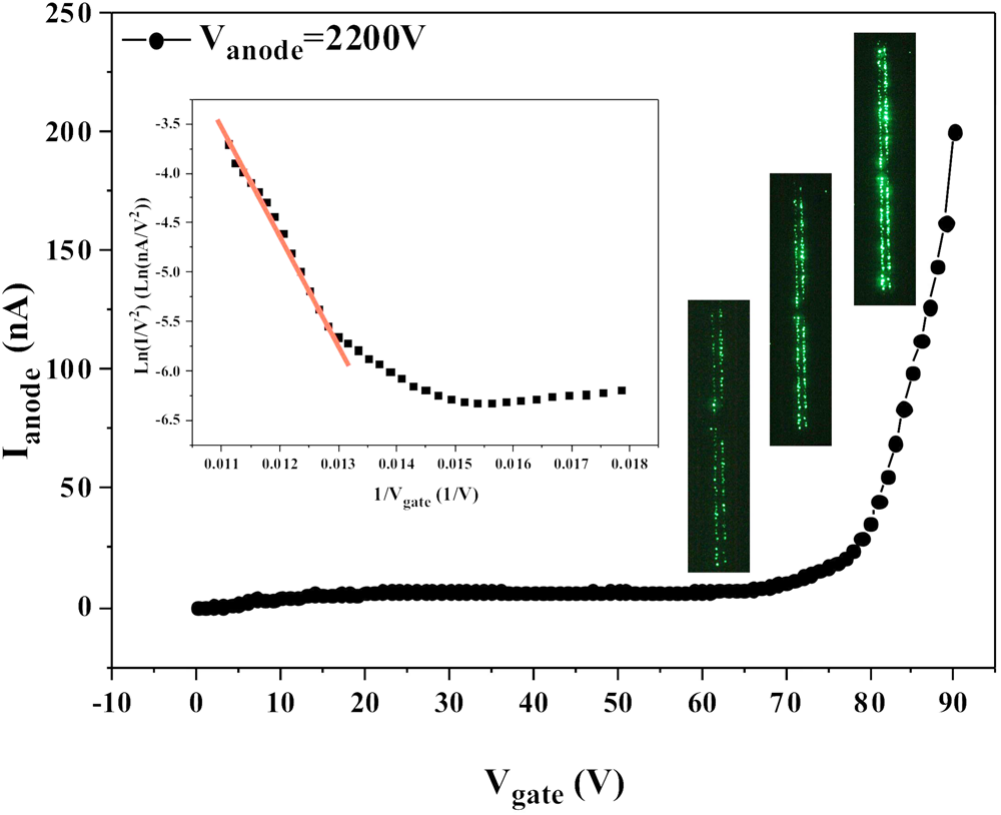
Anode current-gate voltage characteristics of the coplanar-gate ZnO nanowire FEAs, where the inset shows the corresponding F-N plot. Emission images recorded under applied gate voltages of 70 V, 80 V, and 90 V are also superimposed in the plot.

We tested two columns (80 × 2) of the arrays in the FED device. The anode current versus gate voltage curve was recorded under a constant anode voltage, with results as shown in Fig. 5. The inset in Fig. 5 shows the corresponding F-N plot. It is observed that when the gate voltage rises to 70 V, the anode current then begins to increase significantly and reaches up to 200 nA when the gate voltage increases to 90 V. Besides, the field emission images were also recorded under applied gate voltages of 70 V, 80 V, and 90 V, as shown in Fig. 5 and the reproducibility measurement of the gated device was carried out (see Supplementary Fig. S2). Our results indicate that the emission current can be well modulated by the gate voltage and reliable emission can be achieved.

**Figure 6:**
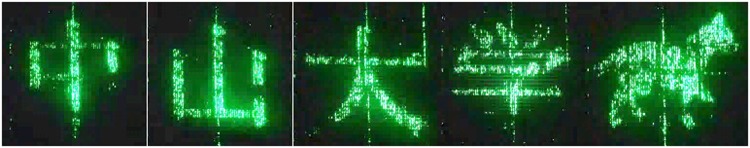
Images showing the device displaying Chinese characters and a cartoon image of a walking dog.

A video driver was then connected to the fabricated device, after an aging process, and stable display of video images was achieved. Figure 6 shows Chinese characters and a cartoon image of a walking dog as displayed using this device. There are 80 × 80 pixels in the display region. The measurements were carried out with an anode voltage of 2000 V, and a peak gate voltage of 100 V. These images verify the addressing capabilities of our coplanar-gate ZnO nanowire FEAs.

## Discussion

**Figure 7:**
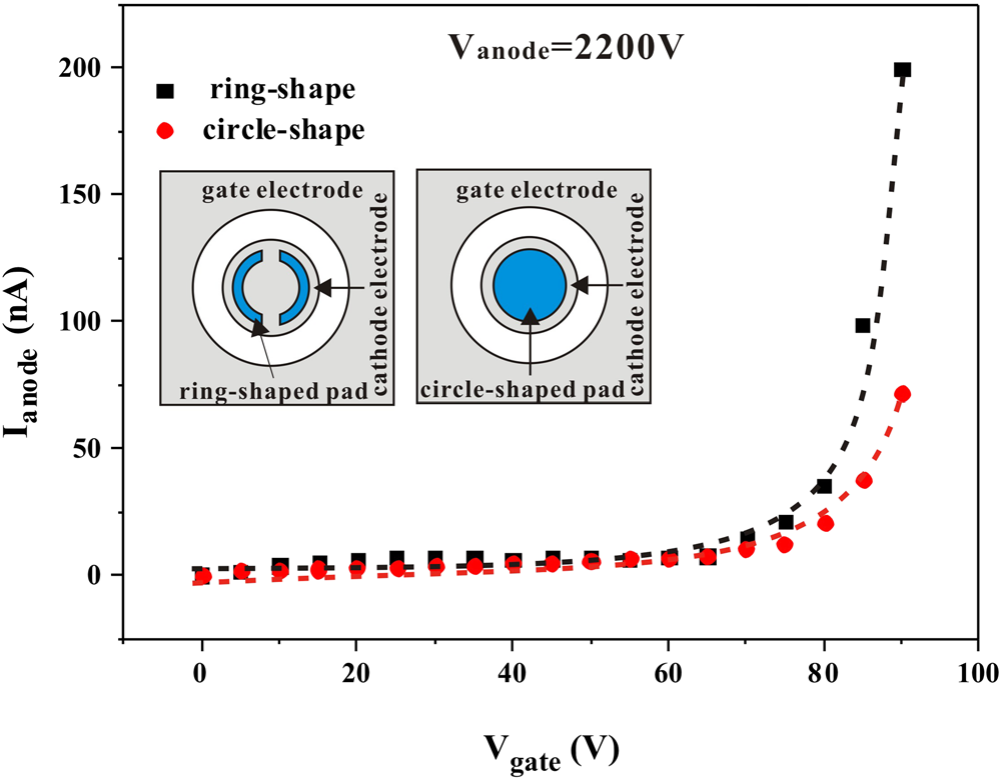
Comparison of the field emission characteristics of coplanar-gate FEAs with ring-shaped ZnO nanowire pads and circle-shaped ZnO nanowire pads.

A comparison of the field emission characteristics of coplanar-gate FEAs with ring-shaped ZnO nanowire pads and circle-shaped ZnO nanowire pads (from our earlier work^29^) is shown in Fig. 7; schematics of the coplanar-gate FEA structures with the ring-shaped ZnO nanowire pads and circle-shaped ZnO nanowire pads are shown in the inset in Fig. 7. This comparison reveals that the ring-shaped ZnO nanowire pads demonstrated better gate-control capability and higher field emission currents. To investigate the origin of this more efficient field emission from the ring-shaped ZnO nanowire pads in coplanar-gate FEAs, we simulated the electric field strength distributions in both the circle-shaped pad and the ring-shaped pad. Based on the simulation results (see Supplementary Fig. S3), when compared with the electric field strength distributions in the circle-shaped pad, the electric field strength at the inner edges of the ring-shaped pad is significantly enhanced. This leads to an increase in the field emission current from the ring-shaped ZnO nanowire pad. In addition, as the gate voltage increases from 0 V to 80 V, while the changes in electric field strength are almost identical in the circle-shaped and ring-shaped pads, the exponential change in the field emission current in the ring-shaped pad is apparently higher than that in the circle-shaped pad because it is based on a higher electric field strength. The simulation results indicate that the regulation of the gate relative to the ZnO nanowires is obviously enhanced because of the increased field strength at the inner edge of the ring-shaped pad.

**Figure 8:**
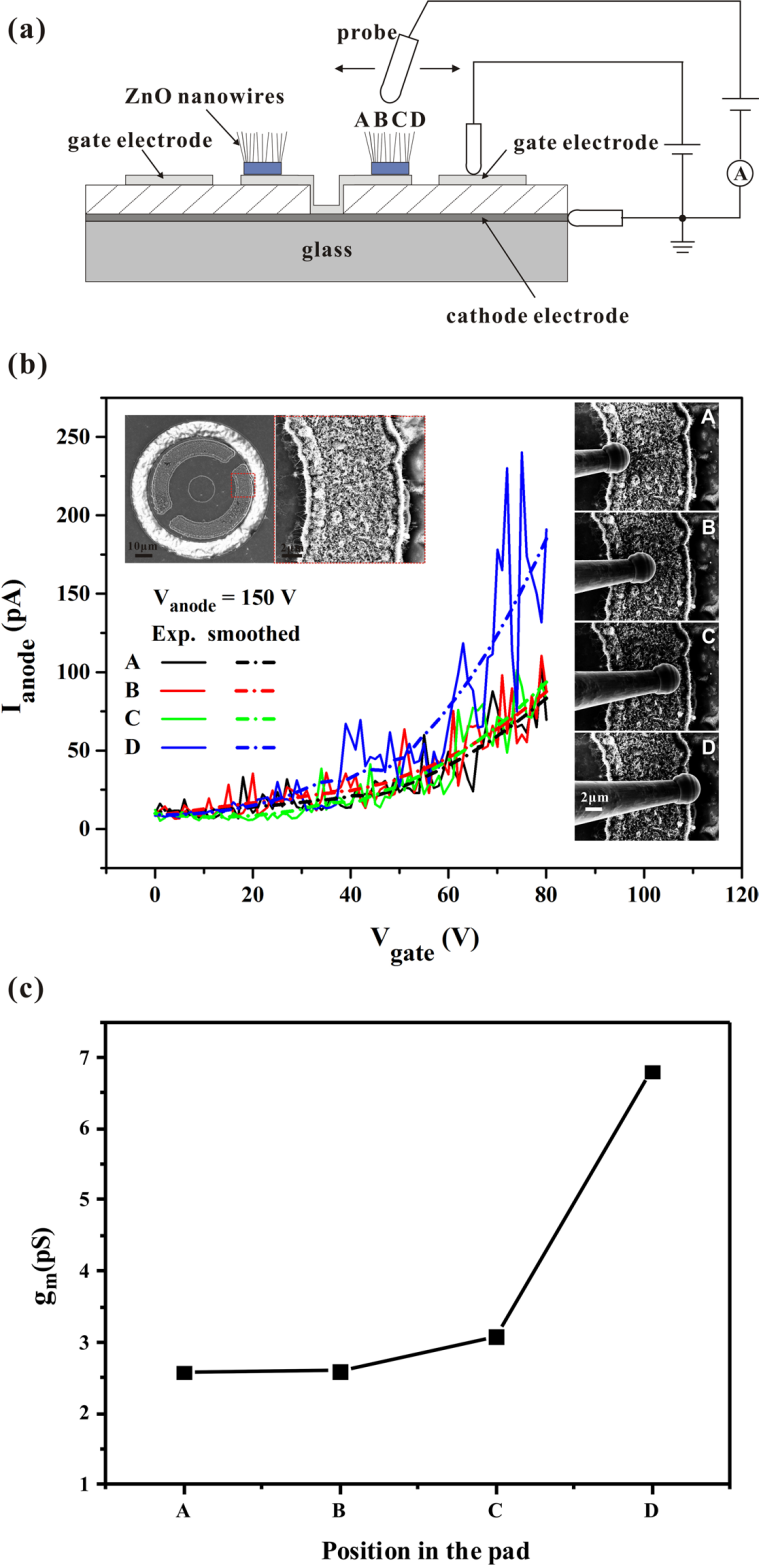
(**a**) Set-up for the microscopic field emission measurements of the ZnO nanowires in the coplanar-gate FEAs; (**b**) field emission currents of the ZnO nanowires in four regions of a single ZnO pad under various gate voltages, where the inset shows the corresponding SEM images; (**c**) transconductance (*g*_*m*_) values of the four regions.

To verify our speculation in this case, microscopic *in-situ* measurements of the field emissions from ZnO nanowires located at different positions within a single ZnO nanowire pad in the coplanar-gate FEAs were performed using a nanoprobe system. The measurement set-up is shown in Fig. 8(a). Two tungsten tips were placed in contact with the gate electrode and cathode electrode, while a third tungsten tip that acted as the anode was moved on top of the ZnO nanowires in various regions with a field emission gap and the emission current was recorded while the gate voltage was varied. The field emission characteristics of ZnO nanowires in four regions of the single ZnO pad are presented in Fig. 8(b). SEM images of the tested ZnO pad in the coplanar-gate FEAs and the four tested regions of the ZnO pad are shown in the inset in Fig. 8(b). The 8-µm-wide half-ring was divided into four regions, which were designated regions A, B, C, and D (where the latter was the closest to the gate electrode). The results show that the field emission currents of these four regions increase with increasing gate voltage, and when the gate voltage increases to 80 V, the currents of regions A, B, C and D are 77, 80, 85 and 190 pA, respectively. The transconductance (*g*_*m*_) is an important parameter that is used to evaluate the ability of the emission current to respond to the gate voltage. The *g*_*m*_ can be expressed as follows:

2 $${{g}_{m}=\frac{d{I}_{anode}}{d{V}_{gate}}|}_{{V}_{anode=const}}$$

At a constant *V*_*anode*_ of 150 V, the estimated *g*_*m*_ values for the four regions are as shown in Fig. 8(c). Region D, which is closest to the gate electrode, shows a higher *g*_*m*_ than the other three regions under the same gate voltage of 80 V. This result implies that the ZnO nanowires in the ring-shaped pad can be regulated using the gate voltage and an enhanced gate control capability is obtained from the ZnO nanowires at the inner edge of the ring-shaped pad because of the increased field strength. It is also noted that the field emission currents from regions B and C should be lower than that from region A according to the simulation results (see Supplementary Fig. S3(b)), but we collected field emission currents from the nanowires in regions B and C that were slightly higher than that in region A in the measurements. We think that the reason for this difference is that the tungsten tip acting as the anode was placed on top of regions B and C during the microscopic measurements, and thus the local fields generated in regions B and C reduced the field-screening effects among the neighboring nanowires in both regions B and C.

In summary, ring-shaped ZnO nanowire pad arrays were integrated into coplanar-gate FEAs and excellent field emission characteristics were obtained from these gated FEAs as a result of the increase edge area by designing ring-shaped ZnO nanowire pad and the suppression of the diode effect. In future work, we intend to narrow the width of the ring appropriately to produce further improvements in the gate-control capability. In addition, we also intend to reduce the distance between the gate and the cathode to reduce the required gate driving voltage.

## Conclusion

A ring-shaped ZnO nanowire pad was proposed and ZnO nanowire pad arrays were prepared by a thermal oxidation method and integrated into gated FEAs. Enhanced gate-control capability was achieved in these coplanar-gate ZnO nanowire FEAs. The coplanar-gate ZnO nanowire FEAs with ring-shaped ZnO nanowire pads presented excellent electron emission characteristics because of the increase edge area by designing ring-shaped ZnO nanowire pad and the suppression of the diode effect. Our work will be helpful in the development of large-area gate-controlled FEAs using nanowire emitters for vacuum electronic device applications.

## Electronic supplementary material

Supplementary Material
