# Supplementary material for: Coplanar-gate ZnO nanowire field emitter arrays with enhanced gate-control performance using a ring-shaped cathode
Source: Sci Rep. 2018 Aug 16;8:12294. doi: 10.1038/s41598-018-30279-y (PMC6095927; doi:10.1038/s41598-018-30279-y)
Supplement: Supplementary file 1 — Supplementary Material [file 41598_2018_30279_MOESM1_ESM.pdf]

Coplanar-gate ZnO nanowire field emitter arrays with enhanced gate-control performance using a ring-shaped cathode

Long Zhao, Yicong Chen, Zhipeng Zhang, Xiuqing Cao, Guofu Zhang, Juncong She, Shaozhi Deng, Ningsheng Xu and Jun Chen*

State Key Laboratory of Optoelectronic Materials and Technologies, Provincial Key Laboratory of Display Material and Technology, School of Electronics and Information Technology, Sun Yat-sen University, Guangzhou 510275, People's Republic of China

*E-mail: stscjun@mail.sysu.edu.cn

S1. The schematic showing the diode effect and explaining the advantage of ring-shaped cathode compared with circle-shaped cathode.

ZnO nanowire field emitters were prepared using a self-assembly method. Therefore, during ZnO nanowire growth, non-uniformity in the length of ZnO nanowires will inevitably occur. If a circle-shaped cathode is used, once some nanowires with large height are formed at the center of the cathode (Figure. S1, the nanowire A), it will not be well controlled by the gate. With increasing anode voltage, these high nanowires will tend to emit electrons without the gate voltage. This is called as "diode-effect". In this case, usually one has to adopt a complicated treatment (or conditioning) process to eliminate the dominating diode emission. This will complicate the fabricate process. While using a ring-shaped cathode, such phenomenon could be avoided.

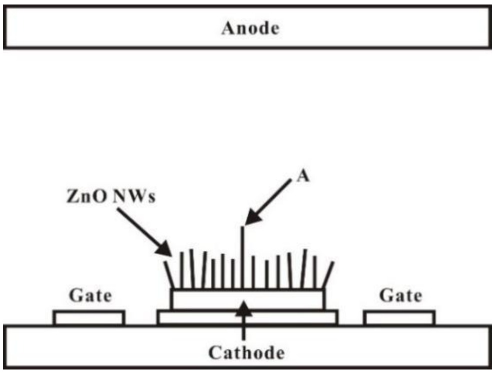

Figure. S1 Schematic showing the diode effect.

S2. The reproducibility measurement of the gated device.

Figure S2 shows the I_{anode}-V_{gate} characteristics obtained from four measurements at different time. It shows that the the characteristics are well repeatable.

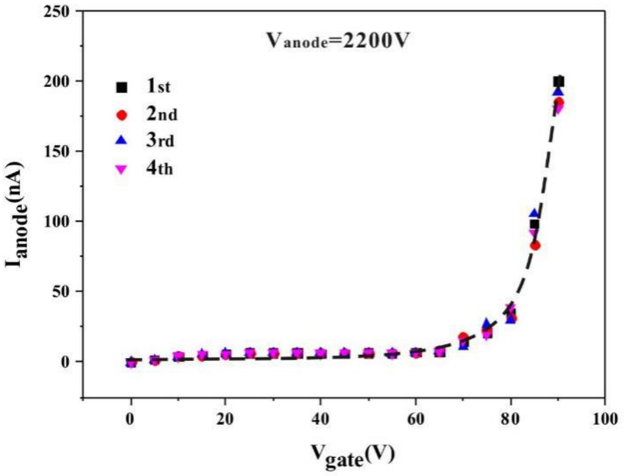

Figure S2 The emission current collected at the anode recorded under different applied gate voltages for four separate measurements. The anode voltage was set at 2200V.

S3. The simulation of electric field strength distributions in circle-shaped pad and ring-shaped pad.

We simulated the distributions of electric field strength at the apex of the nanowires along the cathode in circle-shaped pad and ring-shaped pad using the commercial software COMSOL Multiphysics. A two-dimensional model was adopted by considering the tip morphology of the nanowire. In the simulation, the distance between the anode and cathode plates was 100 μm, the anode voltage was 400 V, the distance between the cathode and gate was 10 μm, and the line widths of the cathode and gate electrodes were 60 μm and 15 μm, respectively.

The simulation results are shown in Figure. S3. In the figure S3(a), it is found that the electric field of the circle is significantly higher at the edge than that at the center when the gate voltage is 0 V, when the gate voltage increases to 80 V, the electric field of the circle at the edge increase rapidly, while the electric field of the circle at the center increase slightly. In the figure S3(b), it is observed that the electric field of the ring are both higher at the outer edge (the closet to the gate electrode) and inner edge than that at the center when the gate voltage is 0 V, when the gate voltage increases to 80 V, the electric field of the ring at the both edges increase, while the electric

field of the ring at the outer edge is higher than that at the inner edge because it is closer to gate electrode.

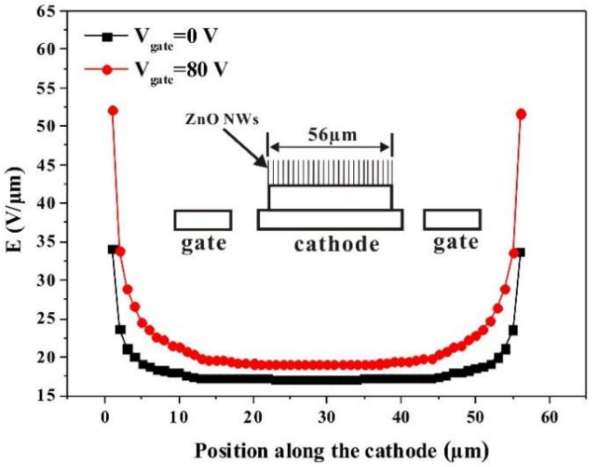

(a)

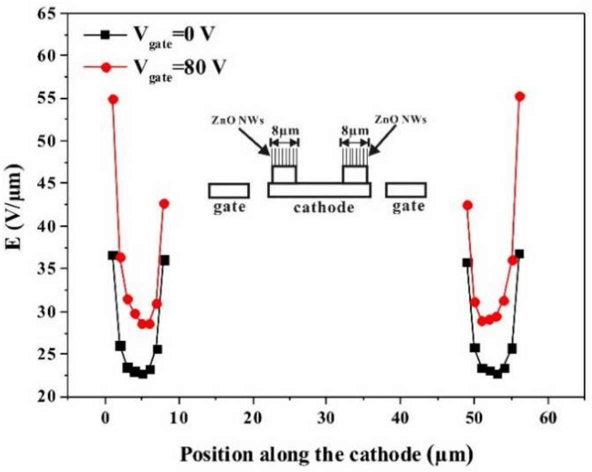

(b)

Figure S3 Simulation results: (a) The electric field strength distribution at the apex of the nanowires along the cathode in the circle-shape pad; (b) the electric field strength distribution at the apex of the nanowires along the cathode in the ring-shape pad.
